# Facile Preparation of Highly Stretchable and Recovery Peptide-Polyurethane/Ureas

**DOI:** 10.3390/polym10060637

**Published:** 2018-06-08

**Authors:** Lin Gu, Yuanzhang Jiang, Jinlian Hu

**Affiliations:** 1 Key Laboratory of Marine Materials and Related Technologies, Key Laboratory of Marine Materials and Protective Technologies of Zhejiang Province, Ningbo Institute of Materials Technology and Engineering, Chinese Academy of Sciences, Ningbo 315201, China; lin.gu@polyu.edu.hk; 2 Institute of Textiles & Clothing, The Hong Kong Polytechnic University, Hong Kong 999077, China; yuan-zhang.jiang@connect.polyu.hk

**Keywords:** polyurethane/urea, poly(*γ*-benzyl-l-glutamate), *β*-sheet conformation, shape recovery

## Abstract

In this work, a new class of highly stretchable peptide-polyurethane/ureas (PUUs) were synthesized containing short *β*-sheet forming peptide blocks of poly(*γ*-benzyl-l-glutamate)-*b*-poly(propylene glycol)-*b*-poly(*γ*-benzyl-l-glutamate) (PBLG-*b*-PPG-*b*-PBLG), isophorone diisocyanate as the hard segment, and polytetramethylene ether glycol as the soft phase. PBLG-*b*-PPG-*b*-PBLG with short peptide segment length (<10 residues) was synthesized by amine-initiated ring opening polymerization of *γ*-benzyl-l-glutamate-*N*-carboxyanhydrides (BLG-NCA), which shows mixed α-helix and *β*-sheet conformation, where the percent of *β*-sheet structure was above 48%. Morphological studies indicate that the obtained PUUs show *β*-sheet crystal and nanofibrous structure. Mechanical tests reveal the PUUs display medium tensile strength (0.25–4.6 MPa), high stretchability (>1600%), human-tissue-compatible Young’s modulus (226–513 KPa). Furthermore, the shape recovery ratio could reach above 85% during successive cycles at high strain (500%). In this study, we report a facile synthetic method to obtain highly stretchable and recovery peptide-polyurethane/urea materials, which will have various potential applications such as wearable and implantable electronics, and biomedical devices.

## 1. Introduction

Nature utilizes an intriguing strategy to yield biomaterials with high performance characteristics, such as stiffness, toughness, and extensibility by exploring hierarchical architecture [1,2]. An examination of natural structural proteins, such as silks, collagens, and elastins, reveals that hierarchical long-range ordered structures of peptides play a critical role in achieving superior mechanical properties [3,4]. For example, spider silks exhibit perfect balance between strength and toughness due to their *β*-sheet, *α*-helical, and random coil structures [5]. Among them, *β*-sheet nanocrystals have been identified as a key component responsible for its excellent mechanical properties [6,7]. By contrast, elastins achieve high stretchability and resilience by covalently crosslinking of short peptide chains [8]. Given their architectural feature and unique properties, the use of structural proteins as building blocks for material design will have tremendous promises in various potential applications such as cell scaffolding, tissue engineering, and smart materials [9]. However, harvesting these natural proteins in large scale is so far difficult.

In recent decades, genetic engineering has been used widely to produce various protein materials with precisely controlled sequences and tailored functions, but this method is costly and inappropriate for mass production [9,10]. Nowadays, chemical synthesis of peptide-based biopolymers to mimic natural proteins has drawn more attention since this process is facile, cost effective, and energy efficient in large scale [9,10]. Recently, Tsuchiya et al. developed multiblock copolypeptides polyAla-*b*-poly(Gly-*r*-Leu) via a two-step chemical synthesis method, which could mimic the secondary structures of dragline spider silks [10]. The Sogah group prepared tetrapeptide (Gly-Ala-Gly-Ala or [Ala]_4_)-containing copolymers, whose mechanical properties were similar to those of regenerated spider silks [11,12,13]. Martino et al. reported the chemical synthesis of cross-linked poly(Orn-Gly-Gly-Orn-Gly), which could mimic almost entirely the physical-chemical properties of elastins, and display similar elastic properties [14].

On the other hand, nature-inspired peptidic segments have been incorporated into polymers to tailor the material properties due to their ability to precisely control second structures [15]. Clarke et al. developed physically cross-linked hybrid hydrogels via grafting *β*-sheet peptides to a poly(*γ*-glutamic acid) backbone [16]. These *β*-sheet peptides provide strong non-covalent cross-links, allowing the hydrogels self-heal after being strained to failure. Hu et al. synthesized shape memory biopolymers containing *β*-sheet polyalanine segments [17]. The *β*-sheet crystals act as netpoints, allowing the biopolymers to exhibit excellent shape recovery ability and high shape fixity. Tanaka et al. prepared well-defined poly(*γ*-benzyl-l-asparate)-*b*-poly(ethylene oxide)-*b*-poly(*γ*-benzyl-l-asparate) (PBLA-*b*-PEO-*b*-PBLA) triblocks, which exhibited excellent strength and flexibility [18]. The extensions could reach 500% from 200% after thermal treatment, which was attributed to a second structural transformation in PBLA, from α-helix to *β*-sheet. Korley et al. utilized peptidic order (specifically *β*-sheets) to design hierarchical polyurethane/ureas [15]. The existence of *β*-sheets made the tensile modulus three-fold increase, but the extension was dramatically reduced. Furthermore, Korley et al. also investigated the effects of second structure and hydrogen-bonding arrangement on the mechanical properties of peptide-polyurea hybrids [19,20]. It was found that increased toughness was attributed to *β*-sheets ordering, and modulus was increased with increased peptide weight fraction.

In this work, a new class of highly stretchable and recovery peptide-polyurethane/ureas (PUUs) were synthesized containing short *β*-sheet forming peptide blocks. The obtained PUUs display medium tensile strength (0.25–4.6 MPa), high stretchability (>1600%), human-tissue-compatible Young’s modulus (226–513 KPa), and high shape recovery ratio (~80%) during successive cycles at high strain (500%), similar to those of elastins. We believe this work will provide new insight for developing highly stretchable polypeptide materials, which would have various potential applications such as wearable and implantable electronics, and biomedical devices.

## 2. Experimental Section

### 2.1. Materials

*γ*-Benzyl-l-glutamate (BLG) and triphosgene were obtained from J & K Scientific Ltd. (Beijing, China). Poly(propylene glycol) bis(2-aminopropyl ether) (PPG-diamine 2000, average *M*_n_ ~2000) was purchased from Aldrich (Beijing, China) and dried at 80 °C in vacuum for 6 h prior to use. Polytetramethylene ether glycol (PTMEG 2000, average *M*_n_ ~2000) was supplied by Shanghai Macklin Biochemical Co., Ltd. (Shanghai, China) and dried at 80 °C in vacuum for 2 h prior to use. Isophorone diisocyanate (IPDI) was purchased from Acros Organics (Beijing, China) and used as received. Tetrahydrofuran (THF) and *N*-dimethylformamide (DMF) were dried over CaH_2_ and distilled before use.

### 2.2. Synthesis of Poly(γ-benzyl-l-glutamate)-b-poly(propylene glycol)-b-poly(γ-benzyl-l-glutamate) (PBLG-b-PPG-b-PBLG) Triblock

**Scheme 1:**
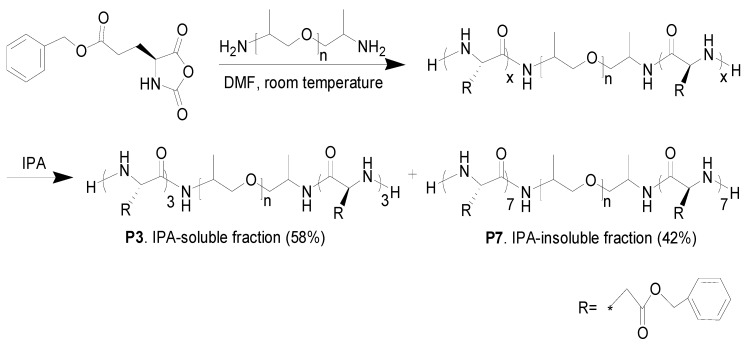
Synthesis of poly(*γ*-benzyl-l-glutamate)-*b*-poly(propylene glycol)-*b*-poly(*γ*-benzyl-l-glutamate) (PBLG-*b*-PPG-*b*-PBLG) triblock.

*γ*-Benzyl-l-glutamate-*N*-carboxyanhydride (BLG-NCA) was prepared from BLG and triphosgene in THF according to the literatures [21,22]. Poly(*γ*-benzyl-l-glutamate)-*b*-poly(propylene glycol)-*b*-poly(*γ*-benzyl-l-glutamate) (PBLG-*b*-PPG-*b*-PBLG) triblock was synthesized by amine-initiated BLG-NCA polymerization, as shown in Scheme 1. In a N_2_ atmosphere, 34.6 g BLG NCA (131.5 mmol), 26.3 g PPG-diamine 2000 (13.15 mmol), and 350 mL DMF were introduced into a flame-dried 1 L flask. The reaction was performed at room temperature for 3 days. The reaction mixture was precipitated into deionized water and filtered. The obtained solid was fractionated by treatment with isopropanol (IPA). The IPA-insoluble fraction was filtered and dried at 50 °C in vacuum to yield **P7** (19 g). The filtrate was dried at 50 °C in vacuum to yield **P3** (27 g). 

### 2.3. Preparation of Peptide-Polyurethane/Ureas and Films

**Scheme 2:**
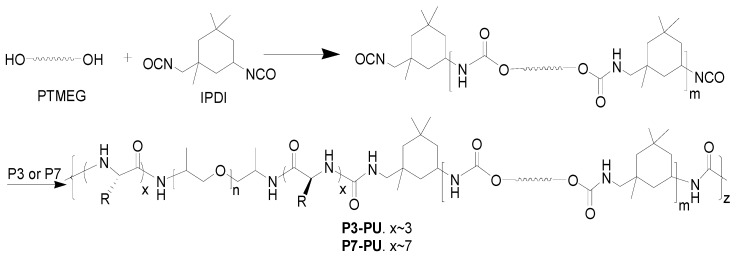
Synthesis of the obtained peptide-polyurethane/ureas (**P3-PU** and **P7-PU**).

The peptide-polyurethane/ureas were prepared via a two-step polymerization process following Scheme 2 according to the literatures [23,24,25], where the molar ratio of PTMEG 2000, IPDI, and peptidic triblock (**P3** or **P7**) was 3:4:1. First, prepolymerization of PTMEG 2000 with IPDI catalyzed by dibutyltin dilaurate was performed at 80 °C for 3 h. Second, the DMF solution of peptidic triblock was added and the reaction was carried out for another 3 h.

The obtained peptide-polyurethane/urea (**P3-PU** or **P7-PU**) was dissolved in DMF to form a 10 wt % solution. The solution was poured into a Teflon mold and dried at 80 °C for 24 h, and then further dried in vacuo at 50 °C for 10 h.

### 2.4. Characterization

^1^H and ^13^C NMR spectra of the obtained peptidic triblocks were obtained in CDCl_3_/CF_3_COOD (3/1, *v*/*v*) using an Avance III 400 MHz spectrometer (Bruker, Billerica, MA, USA). Fourier transform infrared (FTIR) spectra were recorded on a Spectrum 100 FTIR spectrometer (PerkinElmer, Waltham, MA, USA). Differential scanning calorimetry (DSC) analysis was conducted on a DSC 8000 instrument (PerkinElmer, Waltham, MA, USA) under a N_2_ flow from −60 °C to 200 °C at a rate of 20 °C min^−1^. Thermogravimetric analysis (TGA) was performed on a thermal analyzer (METTLER TOLEDO, Greifensee, Switzerland) under a N_2_ flow from 50 °C to 600 °C at a heating rate of 10 °C min^−1^. Wide angle X-ray diffraction (WAXD) data were collected on an X-ray diffractometer (Rigaku SmartLab, Tokyo, Japan) from diffraction angle 2*θ* = 5° to 65° at a speed of 10°/min using Cu Kα radiation (1.54 Å). Atomic force microscopy (AFM) was performed on a scanning probe microscope (Bruker NanoScope 8, Bruker, Billerica, MA, USA).

Tensile test was carried out on an Instron 5566 instrument with a speed of 20 mm/min at room temperature (22 °C). Cyclic tensile test was also conducted on the same machine. First, the sample was stretched to a strain *ε*_m_ at room temperature with a speed of 100 mm/min. Then, the clamp began to return until the force on the sample was 0, and the residual elongation was *ε*_p_. After the above two steps, one cycle is complete. The shape recovery rate (*R*_r_) was calculated according to the following formula (*N* is the cyclic number):

$$R_{r}(N)=\frac{\varepsilon_{m}-\varepsilon_{p}(N)}{\varepsilon_{m}-\varepsilon_{p}(N-1)}\times 100\%.$$

## 3. Results and Discussion

### 3.1. Synthesis and Characterization of Peptidic Triblocks

**Figure 1:**
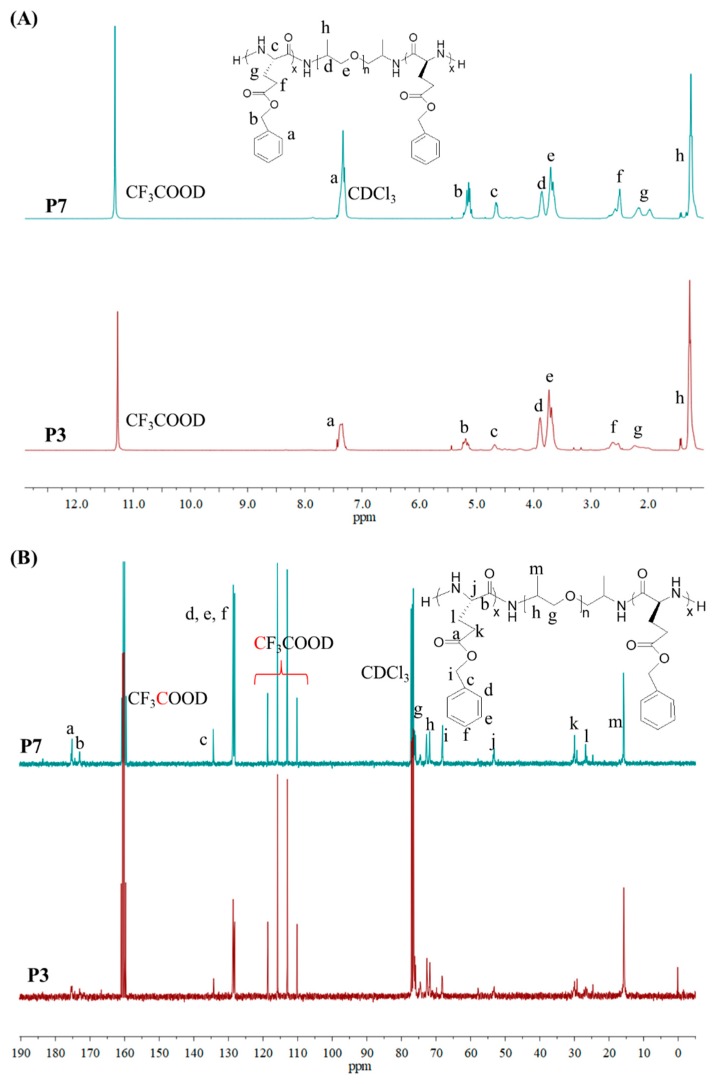
^1^H (**A**) and ^13^C (**B**) NMR spectra of PBLG-*b*-PPG-*b-*PBLG (**P3** and **P7**).

As shown in Scheme 1, PBLG-*b*-PPG-*b*-PBLG was synthesized by amine-initiated ring opening polymerization of BLG-NCA, where the molar ratio of monomer and initiator is 10. The obtained peptidic triblock was fractionated into IPA-soluble (**P3**) and IPA-insoluble (**P7**) components. The structure of these two components was characterized by NMR and FTIR. Figure 1A displays the ^1^H NMR spectra of **P3** and **P7**. The signals at 7.3 ppm and 5.2 ppm ascribed to the protons on phenyl and CH_2_ in benzyl group, respectively, and the characteristic peak at 4.7 ppm is assigned to CH groups in amide linkage. The signals at 1.3, 3.7, and 3.9 ppm are assigned to CH_3_, CH_2_, and CH groups in the PPG units. The molecular weights of **P3** and **P7** were calculated to be ~3314 and ~5066, respectively, according to the integral area ratio of the CH_2_ peak at 5.2 ppm to CH_2_ peak at 3.7 ppm. These correspond to ~6 and ~14 BLG residues, respectively, which are equivalent to an average degree of polymerization (DP) of 3 and 7 for each PBLG block. Figure 1B shows the ^13^C NMR spectra of the samples. The existence of the characteristic peaks of PPG-diamine at 71–76 ppm confirmed the formation of peptidic triblocks.

**Figure 2:**
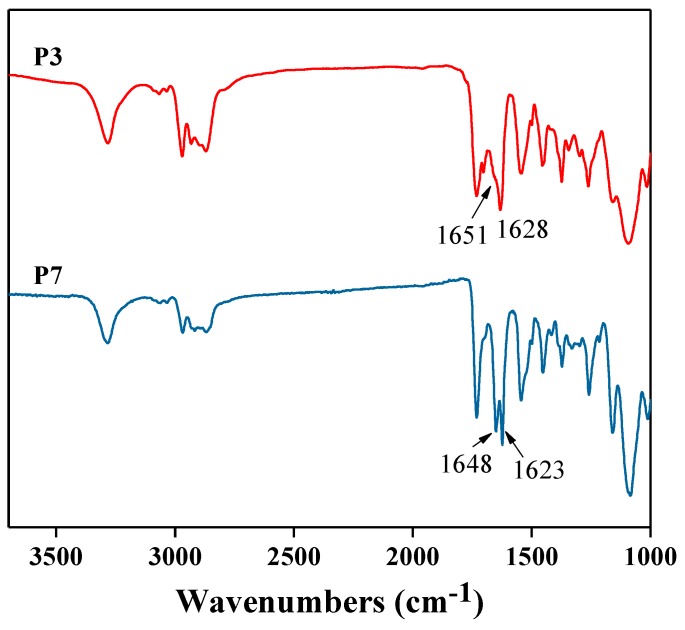
Fourier transform infrared (FTIR) spectra of **P3** and **P7**.

Figure 2 shows the FTIR spectra of **P3** and **P7**. The amide I (C=O stretch) region that is present at 1600–1700 cm^−1^ could be employed to determine the structure (e.g., *β*-sheet, *α*-helix) of polypeptides [21,26]. The strong band of *β*-sheet occurs at 1636–1640 cm^−1^ (parallel) or 1622–1632 cm^−1^ (antiparallel). The amide I peak is located at higher wavenumbers for a left-handed *α*-helix (~1668 cm^−1^) compared to a right-handed *α*-helix (~1655 cm^−1^). Besides, it has been reported that PBLG is more likely to form a *α*-helix confirmation when DP > 10 and a *β*-sheet when DP < 10 [26]. As shown in Figure 2, it can be clearly seen that the obtained **P3** and **P7** not only forms antiparallel *β*-sheet structure, but also has right-handed *α*-helix as previously reported for PBLG-*b*-PDMS-*b*-PBLG [27]. The percent of *β*-sheet conformation in **P3** and **P7** were calculated to be ~83.0% and 48.4% by the deconvolution of the amide I region (1600–1670 cm^−1^), respectively. The shorter the peptide segment PBLG is, the greater is the tendency to form *β*-sheet aggregated.

**Figure 3:**
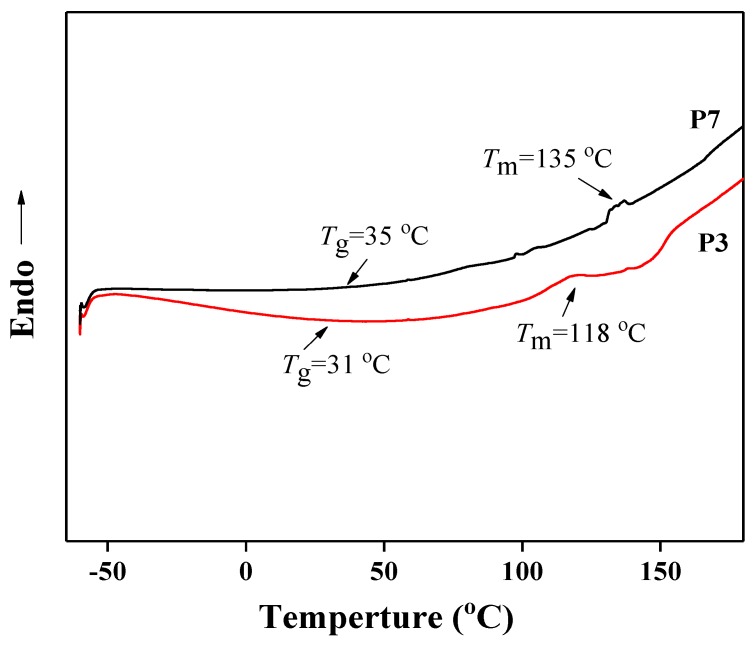
Differential scanning calorimetry (DSC) curves of **P3** and **P7**.

Figure 3 shows the DSC curves of obtained **P3** and **P7**, and their *T*_g_s and *T*_m_s values are listed in Table 1. The glass transition temperatures (*T*_g_s) around 30 °C were detected, corresponding to the PBLG units [28] Moreover, the samples show endothermic peaks at 110–140 °C, which may be attributed to *α*-helical transition [28,29].

### 3.2. Preparation and Properties of Peptide-Polyurethane/Ureas

**Figure 4:**
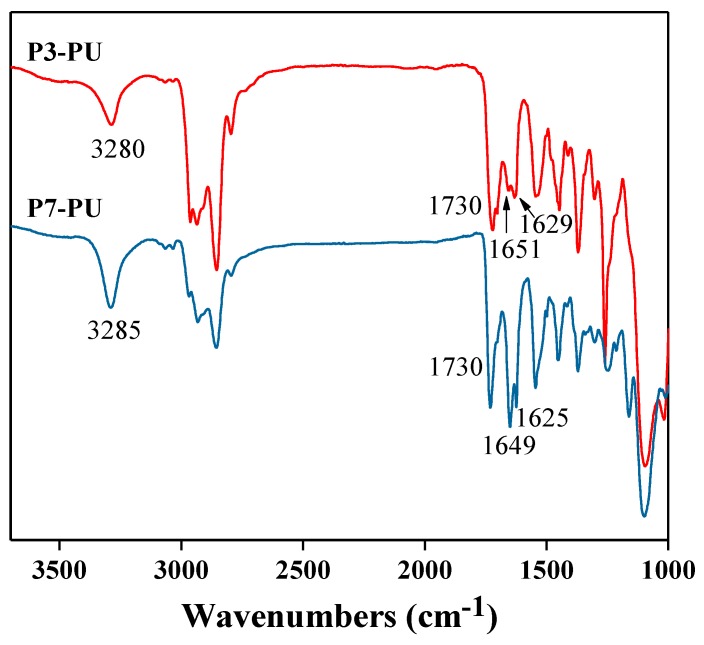
FTIR spectra of **P3-PU** and **P7-PU**.

As shown in Scheme 2, the peptide-polyurethane/ureas (**P3-PU** and **P7-PU**) were prepared using IPDI as the hard segment, PTMEG2000 as the soft phase and PBLG as the peptide segment. The peptide contents in **P3-PU** and **P7-PU** were ~14% and 25%, respectively. FTIR spectra were used to characterize these two samples, as shown in Figure 4. The strong absorptions at ~1730 cm^−1^ are assigned to the stretching vibration of C=O in urethane units and benzyl ester groups, while the peaks at ~1650 cm^−1^ are attituded to the C=O stretching vibration in urea units and amide I regions (*α*-helix). Compared with those of peptidic triblocks, these two peaks become strong, indicating the formation of urethane and urea linkages. Moreover, the strong bands at ~3280 cm^−1^ and 1625 cm^−1^ are corresponding to the N–H stretching vibration and antiparallel *β*-sheet in peptidic triblocks. These results also demonstrate the obtained peptide-polyurethane/ureas have mixed *α*-helix and *β*-sheet structure.

**Figure 5:**
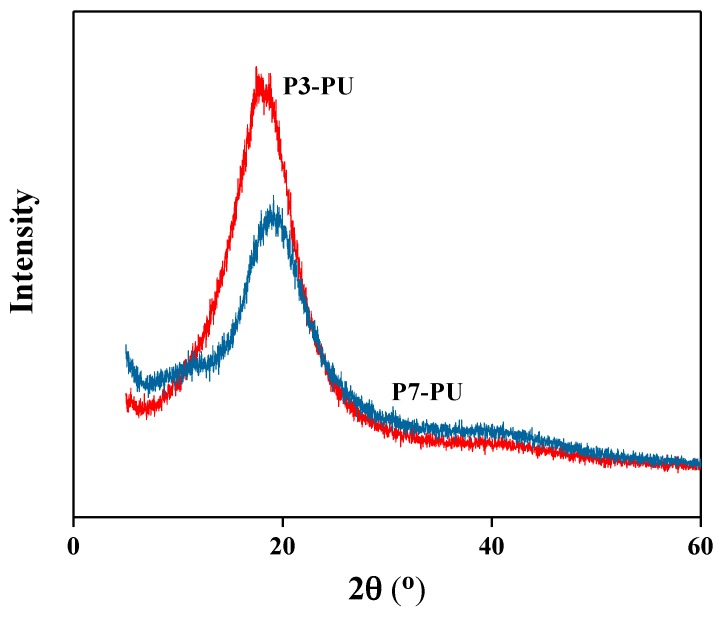
Wide angle X-ray diffraction (WAXD) profiles of **P3-PU** and **P7-PU**.

**Figure 6:**
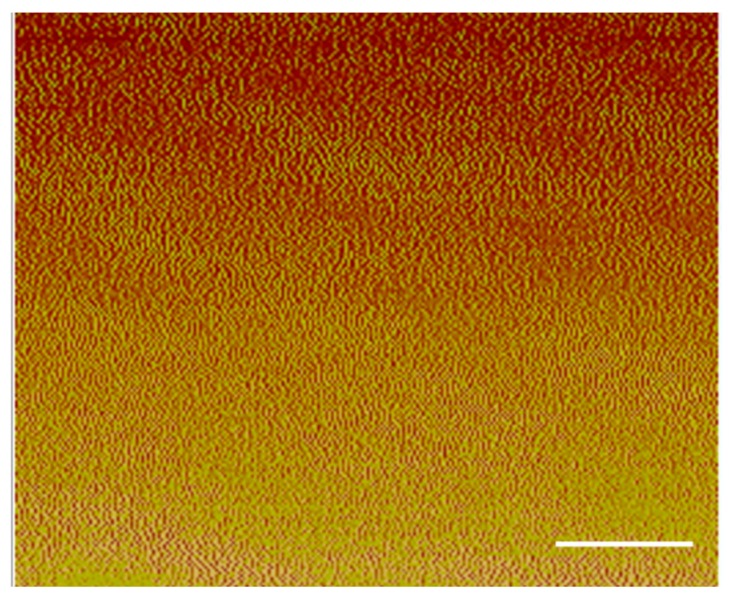
Atomic force microscopy (AFM) phase image of **P7-PU**. The scale bar is 40 nm.

Morphological studies by WAXD and AFM could further elucidate the structural characteristics of the obtained peptide-polyurethane/ureas. Figure 5 exhibits WAXD profiles of **P3-PU** and **P7-PU**. The diffraction peaks at 2*θ* = 17.8° (**P3-PU**) and 19.0° (**P7-PU**) representing the diffraction of (*020*) plane could be ascribed to the *β*-sheet crystalline structure [30]. This indicates that the peptides still exhibit *β*-sheet conformation even upon incorporation into polyurethane/ureas. Moreover, the diffraction peak intensity of **P3-PU** is higher than that of **P7-PU**, which is due to high *β*-sheet content in **P3-PU**. Figure 6 shows the phase morphology of **P7-PU**. Nanofibrous structure was observed in the **P7-PU** film. This was consistent with previously reported peptide-polymer conjugates where hydrogen bonding motifs played a dominant role in the development of microstructures [20].

**Figure 7:**
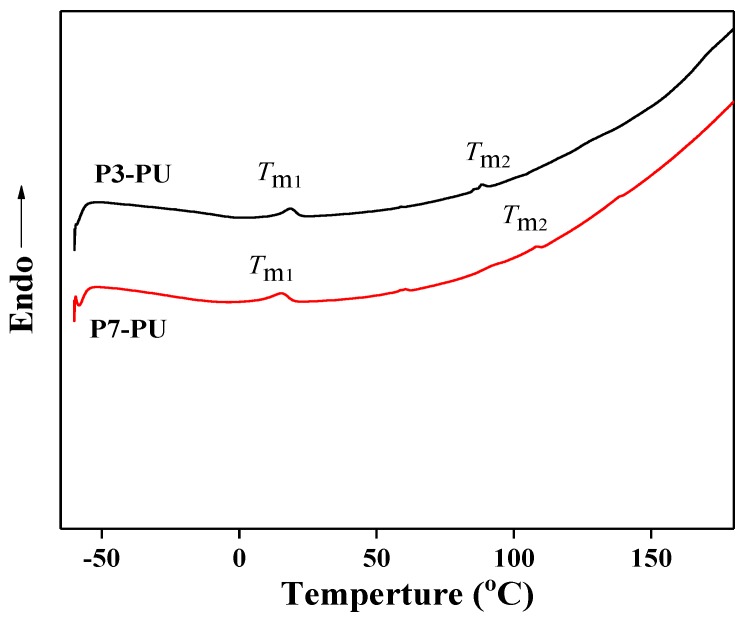
DSC curves of **P3-PU** and **P7-PU**.

**Figure 8:**
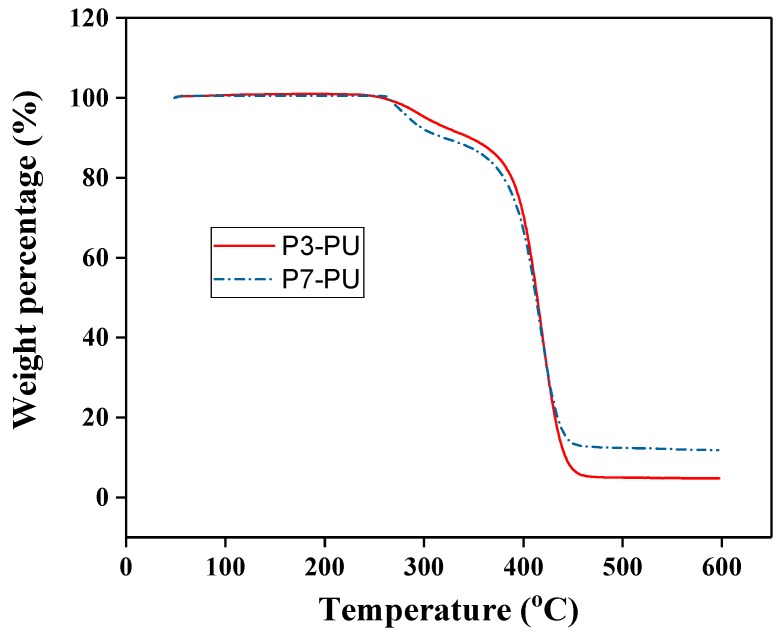
Thermogravimetric analysis (TGA) curves of **P3-PU** and **P7-PU**.

In general, thermoplastic polyurethane/ureas display multiple thermal transitions which correspond to the individual chemical components of the microphase-separated polymer [15]. The thermal transitions of **P3-PU** and **P7-PU** were investigated by DSC. Figure 7 displays the DSC curves of **P3-PU** and **P7-PU** during heating process. Two endothermic peaks were clearly observed, corresponding to PTMEG units (*T*_m1_) and *α*-helical transition (*T*_m2_) in PBLG-*b*-PPG-*b*-PBLG, which were summarized in Table 2. The α-helical transition temperature *T*_m2_ of **P3-PU** and **P7-PU** decreased compared with the pure components, possibly due to the increased mobility induced by the soft PTMEG. Figure 8 shows the TGA results of **P3-PU** and **P7-PU**. The onset decomposition temperature (5% weight-loss temperature) of these two samples was above 280 °C, indicating their good thermal stability.

**Figure 9:**
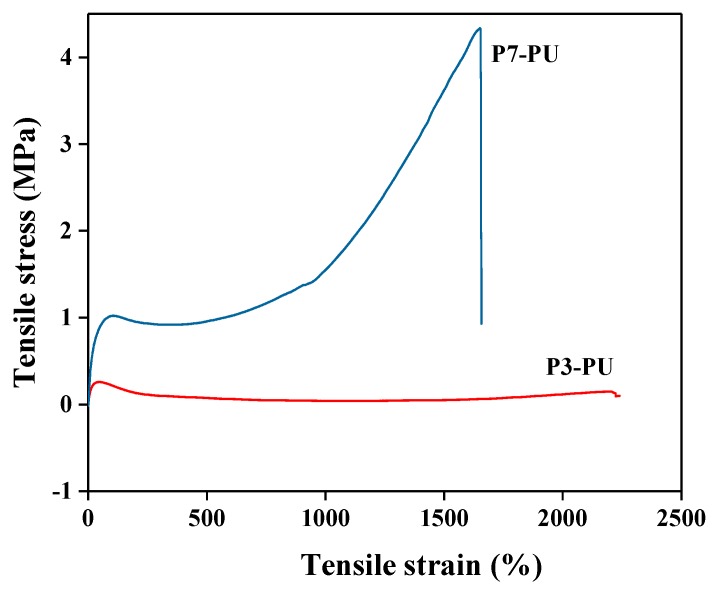
Stress-strain curves of **P3-PU** and **P7-PU**.

**Figure 10:**
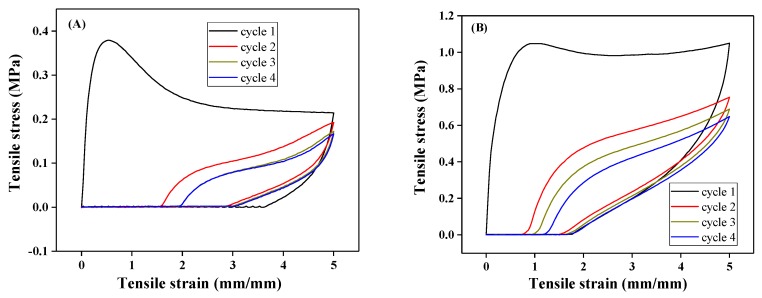
Cyclic tensile curves of **P3-PU** (**A**) and **P7-PU** (**B**) at a strain *ε*_m_ = 500%.

**Figure 11:**
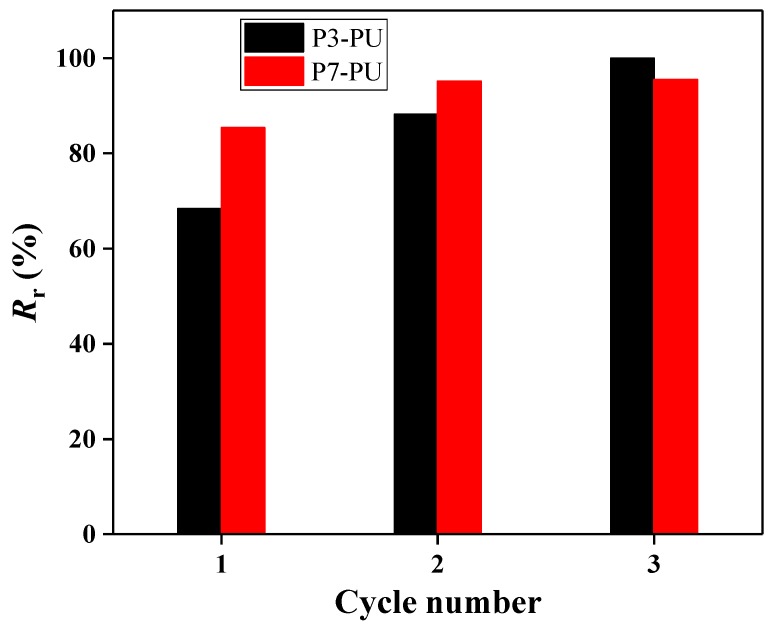
The strain recovery ratio (*R*_r_) during cyclic stretch.

Mechanical properties of **P3-PU** and **P7-PU** were examined by tensile test. Figure 9 shows the stress-strain curves, and their tensile strength, elongation and Young’s modulus are listed in Table 2. They display medium tensile strength (0.25–4.6 MPa), high stretchability (>1600%), human-tissue-compatible Young’s modulus (226–513 KPa), which would have various potential applications such as wearable and implantable electronics, and biomedical devices. Compared to **P3-PU**, **P7-PU** exhibits higher tensile strength and Young’s modulus because of its higher peptide content. It has been reported by that tensile strength and modulus of peptide-polyureas was increased with increased peptide weight fraction, most likely due to the “pseudo” hard segment character of the PBLG blocks [19,20]. Figure 10 shows cyclic tensile curves of the two samples at a strain *ε*_m_ = 500%, and the corresponding values of the shape recovery ratio (*R*_r_) are summarized in Figure 11. It can be found that all the values of *R*_r_ are above 60% during successive cycles at high strain, which may be due to the existing *β*-sheet structures as netpoints [17]. Especially, the *R*_r_ of **P7-PU** is above 85% during the first cycle and >95% after that, indicating good stability in shape.

## 4. Conclusions

PBLG-*b*-PPG-*b*-PBLG with short peptide segment length (<10 residues) was synthesized by amine-initiated ring opening polymerization of BLG-NCA. The obtained peptidic triblocks show mixed *α*-helix and *β*-sheet conformation, and the percent of *β*-sheet structure was above 48%. A new class of peptide-polyurethane/ureas were synthesized containing a peptidic triblock soft segment, which also exhibit both *α*-helix and *β*-sheet structure. The obtained PUUs display medium tensile strength (0.25–4.6 MPa), high stretchability (>1600%), human-tissue-compatible Young’s modulus (226–513 KPa). Furthermore, the shape recovery ratio could reach above 85% during successive cycles at high strain (500%). We believe this work will provide new insight for developing polypeptide materials, which would have various potential applications such as wearable and implantable electronics, and biomedical devices.

## Figures and Tables

**Table 1:** Properties of peptidic triblocks.

Samples	Peptide Content (%)	*β-*Sheet Content (%)	*T*_g_ (°C)	*T*_m_ (°C)
**P3**	39.6	83.0	31	118
**P7**	60.5	48.4	35	135

**Table 2:** Thermal and mechanical properties of peptide-polyurethane/ureas.

Samples	*T*_m1_ (°C)	*T*_m2_ (°C)	Tensile Strength (MPa)	Elongation (%)	Young’s Modulus (KPa)
**P3-PU**	18.6	88.3	0.25	2210	226
**P7-PU**	15.5	108.6	4.69	1650	513
